# Effect of repeated tracheostomy tube reprocessing on biofilm formation

**DOI:** 10.1002/lary.25473

**Published:** 2015-08-12

**Authors:** Jennifer Rodney, Carolyn P. Ojano‐Dirain, Patrick J. Antonelli, Rodrigo C. Silva

**Affiliations:** ^1^ Department of OtorhinolaryngologyUniversity of OklahomaOklahoma CityOklahoma; ^2^ Department of OtolaryngologyUniversity of FloridaGainesvilleFlorida; ^3^ Department of OtolaryngologyUniversity of Florida, University of Florida–Health Shands Hospital for ChildrenGainesvilleFloridaU.S.A

**Keywords:** Bacterial biofilms, tracheostomy tube, effect of reprocessing

## Abstract

**Objectives/Hypothesis:**

To determine the effect of repeated reprocessing of pediatric tracheostomy tubes (TTs) on biofilm formation.

**Study Design:**

In vitro microbiological study.

**Methods:**

Pediatric, uncuffed, polyvinyl chloride (PVC) TTs from two different manufacturers (Tracoe Mini and Shiley) were reprocessed mechanically with household detergent and soaked in sodium hypochlorite (bleach). Two TTs of each brand were reprocessed 0 (control), 10, or 20 times. Twenty 2‐mm coupons were then obtained from each TT, immersed in human mucus, and cultured with either *Staphylococcus aureus* or *Pseudomonas aeruginosa*. Biofilm formation was evaluated with bacterial counts.

**Results:**

Bacterial counts of *S. aureus* for both brands were significantly higher on the TTs that were reprocessed 20 times compared to those that were not reprocessed (Tracoe: *P* = .040, Shiley: *P*  <  .0001) or those that were reprocessed 10 times (Tracoe: *P* = .022, Shiley: *P* = .0002). There was no difference between controls and TTs reprocessed 10 times (Tracoe: *P* = .76, Shiley: *P* = .24). *P. aeuruginosa* counts were not significantly different among the varying numbers of reprocessing cycles for either Tracoe or Shiley TTs (*P* = .08 and *P* = .97, respectively).

**Conclusions:**

Repeated reprocessing of PVC TTs with detergent and bleach paradoxically promotes *S. aureus* biofilm development, possibly due to degradation of the tube surface that facilitates bacterial attachment. Further investigation is needed to determine the optimal technique and limits of reprocessing TTs in clinical practice.

**Level of Evidence:**

NA *Laryngoscope*, 126:996–999, 2016

## INTRODUCTION

Bacterial biofilms remain a common problem associated with indwelling medical devices such as tracheostomy tubes (TTs). They are present on more than 90% of TTs within 7 days of insertion, and standard cleaning methods do not completely remove the bacteria.^1^, ^2^ Biofilms are associated with an increased risk of upper respiratory infections, TT occlusion, and wound infections by *Staphylococcus* and *pseudomonas* spp, among other complications.^3^, ^4^ They can also act as a source of chronic inflammation, leading to a cascade of granulation tissue, bleeding, TT obstruction, and difficulty maintaining airway patency.^5^, ^6^, ^7^

The appropriate length of time until the first TT change has been thoroughly reported in the literature, but the evidence to support chronic TT care practices is sparse. These recommendations are currently based on expert opinion and small observational studies.^8^, ^9^, ^10^, ^11^ No consensus exists regarding the frequency of TT changes in a mature stoma, whether the tube or inner cannula should be cleaned in a specific way or solution, or for how long a tube can be reused safely.^12^, ^13^

We have recently shown that bleach is the most effective cleaning agent in reducing bacterial biofilms on TTs.^14^ However, the effects on the TT material to repeated mechanical reprocessing and exposure to bleach are unknown. We hypothesized that repeated reprocessing could paradoxically promote bacterial biofilm growth as a result of tube surface changes. This study aimed to determine the effects of repeated TT reprocessing on biofilm growth and the number of times a TT can be reprocessed before the susceptibility to biofilm formation increases.

## MATERIALS AND METHODS

A total of 12 TTs of two different brands—Shiley (Tyco Healthcare Group LP, Mansfield, MA) and Tracoe Mini (Bryan Medical Inc., Cincinnati, OH)—were used for this experiment. Two TTs of each brand were reprocessed with household detergent and bleach for either 0 (control), 10, or 20 times. Twenty 2‐mm coupons were obtained from each TT, immersed in human mucus, and cultured with either *Staphylococcus aureus* or *Pseudomonas aeruginosa*. Biofilm formation was evaluated using bacterial counts. The human mucus used in the experiments was collected from patients with Institutional review board approval.

### Tracheostomy Tube Reprocessing and Preparation

Each cycle of reprocessing consisted of washing the exterior of the TT with gauze pads soaked in warm water and a fragrance‐free clear detergent containing sodium laureth sulfate, sodium dodecylbenzene sulfonate, and lauramidopropyl betaine as active ingredients (Palmolive Pure‐Clear; Colgate‐Palmolive Company, New York, NY). Wet gauze pads soaked in the same solution were pulled through the TTs 10 times, and the TTs were then thoroughly rinsed with distilled water. The TTs were then soaked in 0.6% sodium hypochlorite solution (bleach) for 5 minutes. Two TTs from each brand underwent either 0, 10, or 20 reprocessing cycles. Each TT was thoroughly rinsed with distilled water to remove any bleach or detergent residue, and punched to create 2‐mm coupons. The coupons were sterilized using ethylene oxide.

### Bacterial Strains and Biofilm Formation

Frozen aliquots of *P. aeruginosa*, strain Rochester, and *S. aureus* ATCC 29213 were quad‐streaked on tryptic soy agar plates. Three colonies were selected, grown overnight in tryptic soy broth, and placed in an orbital water bath shaker set at 37°C. The bacteria were transferred to a fresh tryptic soy broth and grown to early log phase (optical density of around 0.2 at 640 nm). This yielded approximately 10^8^ colony‐forming units (CFU)/mL of both *P. aeruginosa* and *S. aureus*, as determined by measuring optical density at 640 nm and interpolating the CFU count from a predetermined linear optical density–CFU regression. Twenty sterile coupons of each treatment group—Shiley or Tracoe and 0, 10, or 20 reprocessing cycles—were used for *S. aureus* (n = 120) and *P. aeruginosa* (n = 120). Each coupon was placed in a 96‐well plate, exposed to 200 μL of human mucus for 5 minutes, and allowed to air dry for 5 minutes. The coupons were then incubated with the appropriate bacterial strain at 37°C for 48 hours in the case of *S. aureus* and 96 hours for *P. aeruginosa*. The broth was changed after 24 hours of incubation to prevent nutrient depletion. The coupons exposed to *S. aureus* were treated with 1 mg/mL oxacillin for 24 hours, and those containing *P. aeruginosa* were treated with 200 μg/mL gentamicin for 24 hours to kill planktonic bacteria.

### Biofilm Analysis

Each coupon was rinsed with 200 μL of 1 molar phosphate‐buffered saline (PBS) four times for 5 minutes each, and placed into a 15‐mL flip‐top conical tube (Thermo‐Fisher Scientific, Rochester, NY) containing 2 mL sterile PBS containing five parts per million Tween‐80 (Fisher Chemical, Fair Lawn, NJ). The samples were then sonicated (Branson Ultrasonics, Danbury, CT) five times for 1.5 minutes with a 1‐minute rest in between, and subsequently vortexed. The samples were then serially diluted and plated in triplicates on tryptic soy agar plates. These plates were incubated for 18 to 24 hours, and the colonies were counted manually. The number of colonies was used as an indirect measure of the presence of biofilms.

### Statistical Analysis

Statistical analysis was performed using JMP 8.0 statistical software (SAS Institute Inc., Cary, NC). Bacterial counts among all study groups were compared using analysis of variance, followed by the Student *t* test, if the overall test result was significant. The confidence level was set for 95% for all tests.

## RESULTS

**Figure 1:**
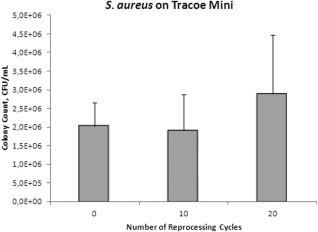
Mean *Staphylococcus aureus* (*S. aureus*) colony counts in colony‐forming units (CFU) per milliliter in Tracoe Mini tubes after different numbers of reprocessing cycles. Error bars indicate standard deviation.

**Figure 2:**
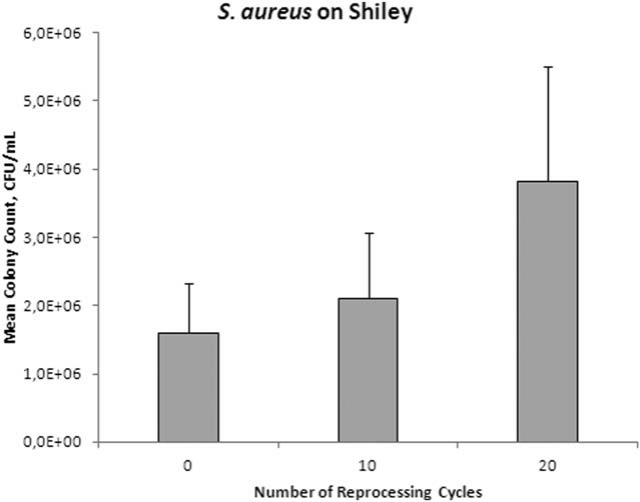
Mean *Staphylococcus aureus* (*S. aureus*) colony counts in colony‐forming units (CFU) per milliliter in Shiley tubes after different numbers of reprocessing cycles. Error bars indicate standard deviation.

**Figure 3:**
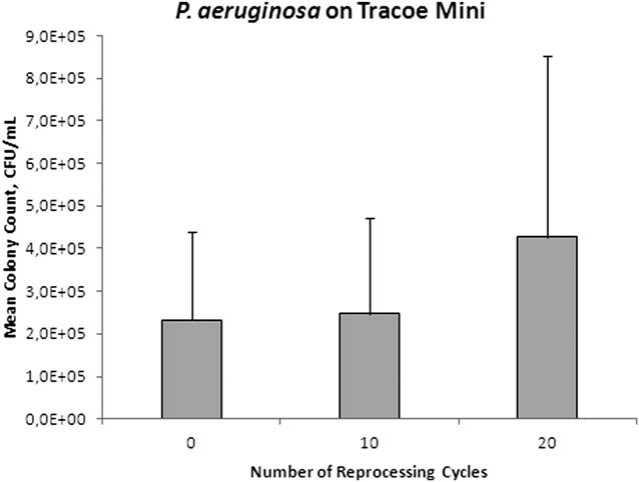
Mean *Pseudomonas aeruginosa* (*P. aeruginosa*) colony counts in colony‐forming units (CFU) per millimeter isolated from Tracoe Mini tubes after different numbers of reprocessing cycles. Error bars indicate standard deviation.

**Figure 4:**
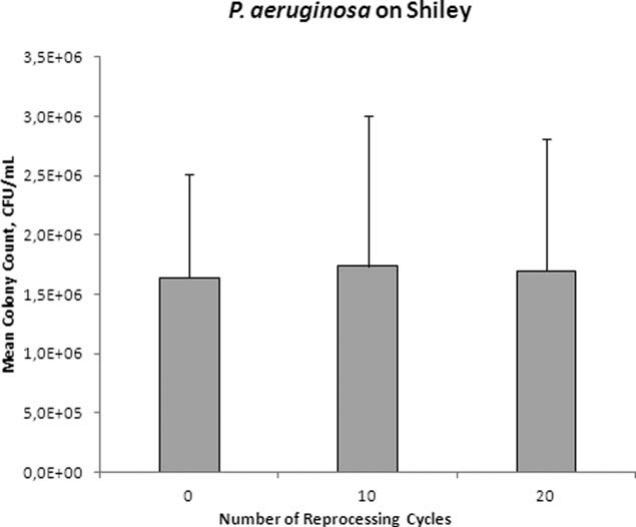
Mean *Pseudomonas aeruginosa* (*P. aeruginosa*) colony counts in colony‐forming units (CFU) per milliliter (mL) in Shiley tubes after different numbers of reprocessing cycles. Error bars indicate standard deviation.

After reprocessing the TTS 0, 10, or 20 times followed by 2‐day exposure to *S. aureus,* bacterial counts of *S. aureus* for both brands were significantly higher in the TTs that were reprocessed 20 times compared to those that were not reprocessed (Tracoe: *P* = .040, Shiley: *P*  <  .0001) or those that were reprocessed only 10 times (Tracoe: *P* = .022, Shiley: *P* = .0002). There was no significant difference in bacterial counts between TTs that were not reprocessed compared to TTs that were reprocessed 10 times (Tracoe: *P* = .76, Shiley: *P* = .24; Figs. 1 and 2). *P. aeruginosa* counts were not significantly different between the varying numbers of reprocessing cycles for either Tracoe or Shiley TTs (*P* = .08 and *P* = .97, respectively; Figs. 3 and 4).

## DISCUSSION

This is the first study, to our knowledge, that measures the effect of TT reprocessing on biofilm formation. Twenty cycles of reprocessing resulted in a significant increase in growth of *S. aureus* biofilm compared to the TTs that were reprocessed less. *P. aeruginosa* biofilm was not significantly affected by TT reprocessing, though the bacterial counts tended to be higher in the Tracoe TTs reprocessed 20 times compared to those that were reprocessed 10 times or not reprocessed at all. Both detergent and bleach were used to reprocess the TTs due to previous evidence that bleach provided additional benefit compared to other cleaning solutions.^14^

Despite countless advances in management of bacterial infections, bacterial biofilms remain a difficult problem. Biofilms are complex systems with channels for nutrient and water exchange. They have been proven to survive despite adequate activation of the humoral and cellular immune response and antibiotic treatment because of the resistant exopolysaccharide matrix coating, lower metabolism, and sharing of resistance genes. TTs are particularly susceptible to biofilm formation because of the inherent skin barrier breakdown and subsequent airway colonization, as well as exposure to substrate that may enhance bacterial binding, such as respiratory mucus and blood.^3^, ^15^, ^16^ The implications for tracheostomy patients may involve increased formation of granulation tissue, local infections, and TT occlusion.

To avoid these complications, maintaining a “clean” TT and stoma is usually recommended by most clinicians. However, little research has been done to determine how many times a TT should be reprocessed and when it should be discarded. Previous research showed that TTs have significant signs of degradation after 3 months of use, and that biofilm was noted to be more prevalent on the TTs after 3 months compared to 1 month of use.^17^ Although surface wear varies according to TT material and cleaning solution used, it seems prudent to recommend that each TT should not be cleaned more than 10 times, and not be used longer than 3 months, before the susceptibility to bacterial biofilm formation starts to increase.

The differences observed between *S. aureus* and *P. aeruginosa* are most likely related to the coating of the samples in human mucus before exposure to the different bacteria. Mucins of the tracheobronchial tree have been shown to be preferential sites for adherence and colonization by *P. aeruginosa*.^18^ We hypothesize that the conditions for *P. aeruginosa* adherence were already maximized by the coating of the surface with mucus, and were not affected by the changes in the polyvinyl chloride (PVC) caused by multiple cycles of reprocessing.

Limitations of this study include the inherent differences of an in vitro experiment compared to an in vivo environment. Human mucus was used to coat the TT coupons before bacterial incubation in an effort to minimize this difference. Other elements of an in vivo tracheal environment include the constant airflow and other colonizing bacteria that were not included in this model. Both brands of TT used were made of PVC, which may limit the generalizability of the results. In addition, we utilized standard microbiological technique and bacterial colony counts as a measure of total biofilm burden on each surface. Although colony counts have been an established method to quantify the biofilms on different surfaces in vitro and in vivo, other optical and molecular biology methods, such as fluorescence in situ hybridization in conjunction with confocal microscopy, could complement our findings as a second quantitative method, and also allow the analysis of the three‐dimensional structure of the biofilm and staining for bacterial vitality.^19^

## CONCLUSION

Our results demonstrate that repeated reprocessing apparently alters the TTs such that *S. aureus* biofilms more readily grow on the TT surface. Future research should focus on more biofilm‐resistant materials or cost‐effective disposable tubes.
